# Intercalary Reconstructions with Vascularised Fibula and Allograft after Tumour Resection in the Lower Limb

**DOI:** 10.1155/2013/160295

**Published:** 2013-05-23

**Authors:** Katharina Rabitsch, Werner Maurer-Ertl, Ulrike Pirker-Frühauf, Christine Wibmer, Andreas Leithner

**Affiliations:** Department of Orthopaedic Surgery, Medical University of Graz, Auenbruggerplatz 5, 8036 Graz, Austria

## Abstract

Reconstruction with massive bone allograft and autologous vascularised fibula combines the structural strength of the allograft and the advantages of fibula's intrinsic blood supply. We retrospectively analysed the outcome of twelve patients (4 male, 8 female) who received reconstruction with massive bone allograft and autologous vascularised fibula after tumour resection in lower limb. Mean age was 17.8 years (range 11–31 years), with following primaries: Ewing's sarcoma (*n* = 6), osteosarcoma (*n* = 4), liposarcoma grade 2 (*n* = 1), and adamantinoma (*n* = 1). Mean followup was 38.7 months (median 25.7 months; range 2–88 months). Seven tumours were located in the femur and five in the tibia. The mean length of bone defect was 18.7 cm (range 15–25 cm). None of the grafts had to be removed, but there occurred four fractures, four nonunions, and two infections. Two patients developed donor side complication, in form of flexion deformity of the big toe. The event-free survival rate was 51% at two-year followup and 39% at three- and five-year followup. As the complications were manageable, and full weight bearing was achieved in all cases, we consider the combination of massive bone allograft and autologous vascularised fibula a stable and durable reconstruction method of the diaphysis of the lower limbs.

## 1. Introduction 

Limb salvage has become the primary method of bone tumour treatment due to improved therapeutic options, combining polychemotherapy, wide resection, and case-based additional radiation. This led to improved prognosis, with 5-year survival rates up to 85% and 10-year survival rates up to 75% for these patients [1–7]. Advances in diagnostic imaging techniques permit an accurate preoperative determination of the tumour extent [8]. If this tumour extension allows preservation of adjacent joints and intercalary resection, the functional outcome is expected to be superior in intercalary reconstructions, since no joint replacement—neither prosthetic nor allograft—could function better than an intact native joint [9–13]. Intercalary defects can be reconstructed by using massive bone allografts [8, 14–23], vascularised autologous fibula grafts [24–28], the combination of both [27, 29–39], nonvascularised fibula grafts [40, 41], and intercalary prostheses [42–46].

Reconstruction with a massive bone allograft and an autologous vascularised fibula combines the structural strength of the allograft and the advantages of fibula's intrinsic blood supply [30]. To assess the postoperative outcome in our patients, we evaluated durability and complication rates.

## 2. Material and Methods

We identified twelve patients (4 male, 8 female) who received an autologous vascularised fibula for reconstruction of seven femoral and five tibial defects at the Department of Orthopaedic Surgery, Graz Austria. Patients' medical records were scanned for the following data: age at operation, length of followup, pathology and localisation of the tumour, additive treatment with chemotherapy or radiation, length of bone defect, fixation device, operation time, time to partial weight bearing and time to full weight bearing, complications due to the allograft (infection, fractures, and nonunion) as well as complications due to the tumour (local recurrence, distant metastases, and death of disease), revision procedures, and failure of the reconstruction, defined as removal of the construct for any reason. 

Descriptive statistics included means and proportions depending on the type of data. The survival rates of patients as well as of reconstruction were estimated using the Kaplan-Meier method. Log-rank test was used to compare survival curves. Pearson's chi-square test and two-tailed Fisher's exact test were used to analyze correlations. 

## 3. Results

### 3.1. Patients and Reconstructions

Twelve patients received an autologous vascularised fibula combined with a massive allograft to reconstruct a segmental defect after tumour resection with a mean followup of 38.7 months (median 25.7; range 2–88 months). There were eight female and four male patients with a mean age of 17.8 years (median 14.3 years; range 11–31 years) at time of reconstruction. Seven tumours were located in the femur and five in the tibia. Primary diagnoses included six Ewing's sarcoma, four osteosarcoma, one liposarcoma grade 2 (grading according to the American Joint Committee on Cancer/International Union Against Cancer (AJCC/UICC) staging system [47]), and one adamantinoma. Four patients underwent wide resection only; eight patients received additional multimodal treatment including chemotherapy in six patients, chemotherapy and radiation in one patient, and additional radiation only in one patient. 

The mean length of bone defect was 18.7 centimetres (median 18.8 cm; range 15–25 cm). The graft-host junctions were augmented with autologous cancellous bone grafts at primary surgery in one patient, while eleven patients had no additional bone grafting. On average the surgical procedure lasted 232 minutes (median 115 minutes; range 86–723 minutes). 

### 3.2. Oncologic Results

At latest followup nine patients were alive without evidence of disease, and none of the twelve patients presented local recurrence. Three patients developed distant metastases in the lung at an average of 10 months after primary surgery—two with Ewing's sarcoma and one with osteosarcoma—and, despite metastasectomy and chemotherapy, died of disease.

**Figure 1:**
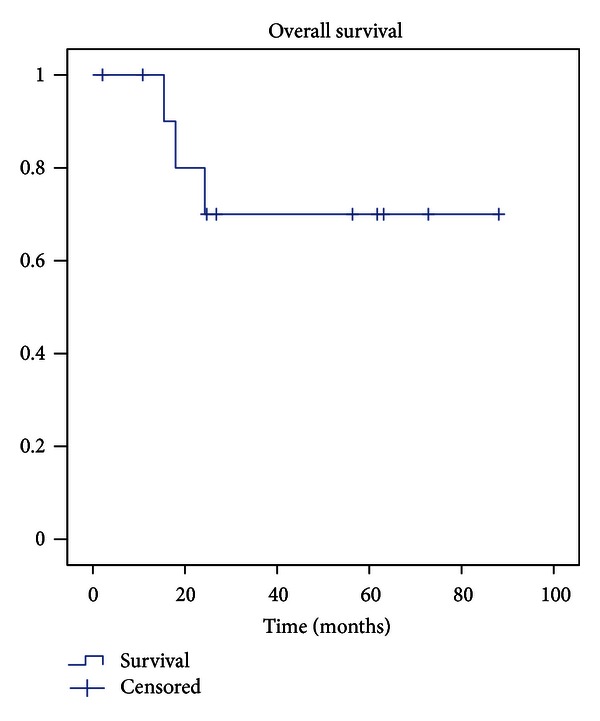
Kaplan-Maier curve for patients' overall survival rate with death of disease as endpoint.

The overall survival rate for all patients was 80% after two years and 70% after three and five years (Figure 1). 

### 3.3. Graft Survival and Complications

All patients achieved full weight bearing without support of braces, crutches, or a cane, and none of the grafts had to be removed. Partial weight bearing was allowed at two months after operation on average (median 1.7 months; range 1–4.6 months) and full weight bearing at a mean of 9.4 months after operation (median 7.9 months; range 3.7–27.6 months). The longest period of load relief was 28 months in a patient with nonunion and a fracture. 

**Figure 2:**
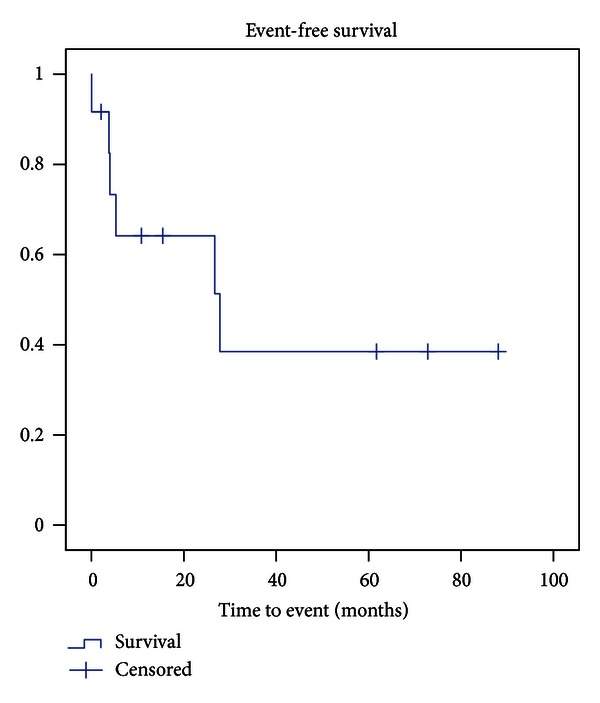
Kaplan-Maier curve for event-free survival, with event defined as any complication requiring additive surgical intervention.

Six patients had 14 additional surgical interventions due to complications at 10.8 months (mean) after primary surgery on average. The event-free survival rate (Figure 2), with event defined as any complication requiring an additional surgical intervention, was 64% after two years and 39% after three and five years.

**Figure 3:**
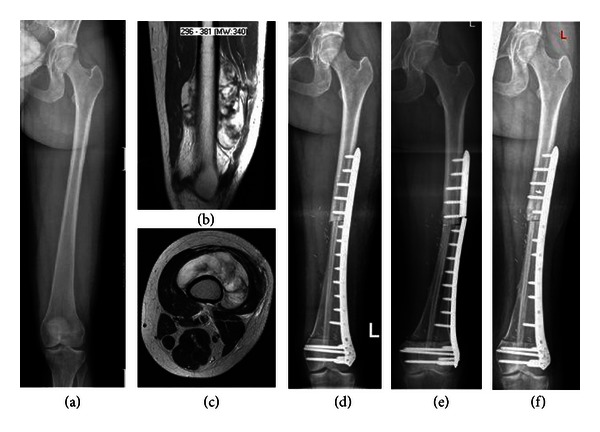
X-ray and MRI imaging in a 31-year-old female patient with Liposarcoma grade 2 of the left femur: before operation (a)-(b), one week after operation (c), plate breakage and fracture 15 months after operation (d), and one month after revision (e).

Seven patients had thirteen local complications at 11 months after primary surgery on average. Four patients, all with femoral reconstructions, sustained graft fracture. Two of them had an adequate trauma: one patient fell because one of his crutches broke in early mobilisation period, and the second patient fell off her bicycle before graft host junctions had totally healed. In the other two patients nonunion or delayed union of the proximal graft-host junction led to wearing of the plate and subsequent plate and graft fracture (Figure 3). All four fractures were successfully treated with open reduction, replacement of internal fixation, and perifractural augmentation with autologous iliac cancellous bone grafts. Four of seven femoral reconstructions fractured, while no tibial reconstruction sustained a fracture (*P* = 0.038). No tendency for higher risk for fracture could be seen anent length of bone defect, time to partial weight bearing, or time to full weight bearing in this analysis (Pearson's chi-square test: *P* = 0.544; *P* = 0.819; *P* = 0.477).

**Figure 4:**
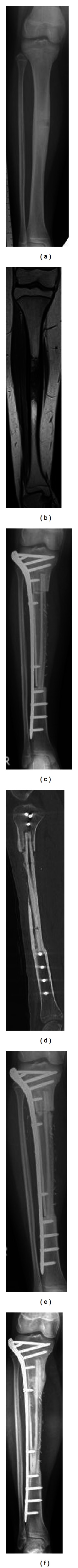
X-ray, CT and MRI imaging in a 14-year-old female patient with Ewing's sarcoma of the right tibia: before operation (a)-(b); three months after operation (c), seven months after operation: no consolidation at the proximal graft host junction (d)-(e); 21 months after operation: fully integrated fibula; and allograft (f).

Four patients presented nonunion, two leading to subsequent fracture, as mentioned above. One case of nonunion was successfully treated with ESWT (Extracorporeal Shock Wave Therapy) and one healed without further intervention 18 months after primary surgery (Figure 4).

Wound healing disorders occurred in two patients, both in tibial reconstructions. They both were treated with debridement, vacuum-assisted wound closure (necessary in one patient) and consecutive wound coverage. One of the patients with wound healing disorder developed deep infection which was successfully managed with removal of internal device and systemic antibiotics. Another patient also sustained local infection but died due to progressive disease before treatment of infection could be started. Haematoma requiring surgical intervention occurred in one patient.

A trend could be identified that tibial reconstructions favours wound healing disorders, since all occurred in tibial reconstructions (*P* = 0.067). 

Two patients had donor side complications, both developed flexion deformity of the big toe. 

## 4. Discussion

Due to more accurate imagine techniques and improved multimodal treatment concepts intercalary resection without compromising safe wide resection margins is possible even if only little juxtaarticular bone is tumour free. In children and adolescent patients preservation of the epiphyseal segment affords further growth. If only a part of the epiphysis is preserved, the risk of varus or valgus deformities rises [9–13].

One popular solution for reconstruction of intercalary defects secondary to tumour resection is the use of massive allografts. Intercalary allografts have a good reputation as better results can be achieved compared to other allograft reconstructions, like osteoarticular allografts or composite allograft reconstructions [9, 17, 48–52]. If there is access to a bone bank, allografts can be obtained—in different sizes and lengths. Massive allografts preserve bone stock, allow adequate attachment of salvaged tendons, and provide initial mechanical strength [14, 16, 17]. After healing, the graft may be progressively incorporated by the host, and it has been demonstrated that intercalary allografts can survive for decades. Ten-year graft survival rates of approximately 80% had been reported (range 53% to 84%) [8, 14–16, 18]. Long-term results of several study groups report a steady state without deterioration of the graft if the allograft endures the first three years, suggesting that it will thereafter remain functional for the duration of patient's life [9, 11, 12, 16, 18, 22, 49–51, 53, 54]. Good functional long-term results are achieved with intercalary allografts, but associated complications as nonunion, fracture, and infection are frequent (Table 1). Up to 70% require additional surgical interventions due to complications, and it has been seen that occurrence of one event of the triad “infection, fracture, and nonunion” compromises the final outcome [17, 48–51, 53, 55, 56]. These problems are the consequence of the graft's avascular status and the incomplete revascularisation and incorporation by the host [9, 30, 32, 36, 57, 58].

To reduce these complications, and improve the outcome, the massive allograft can be combined with an autologous vascularised fibula. The allograft supplies initial mechanical strength, and the fibula provides well-perfused bone and the capability of osteogenesis [9, 13, 30–32, 34, 35]. During the first years the allograft supports the narrow and weak fibula mechanically, but it does not totally shield the fibula from weight bearing. This exposure to weight bearing induces a progressive concentric fibular hypertrophy. Due to this hypertrophy the fibula can compensate weakening of the graft by creeping substitution, the process of vascularisation, resorption and replacement of the graft's scaffolding with new host bone, what leads theoretically to a lower fracture risk [9, 20, 31, 59]. We observed four fractures in twelve patients. Two of them had an adequate trauma causing the fracture, both in a relatively early mobilisation period, when neither union nor hypertrophy of the fibula was completed. The other two fractures happened subsequently to nonunion, plate wearing, and subsequent plate breakage. The well-perfused fibula is advantageous in the treatment of fractures: all four fractured grafts healed similar to normal bone fractures after open reduction, replacement of internal fixation, and autologous bone grafting. Similar observations concerning fracture healing in combined grafts—unless the anastomosis fails—are reported in the literature (Table 2) [32, 34, 35, 55].

The osteogenic potential of the vascularised fibula does not only improve fracture healing but allows a more rapid and reliable fusion between graft and host, reducing the risk of nonunion. A review of literature reveals lower nonunion rates for combined allograft and vascularised fibula reconstructions (up to 31%) in comparison to allograft alone (nonunion in up to 46%) (Tables 1 and 2) [11, 12, 14, 20, 22, 23, 27, 30–38, 54]. Among our twelve patients, four developed nonunion. One of them has to be seen as a delayed union, since the junction healed without further treatment after 18 months. In one patient, the nonunion was successfully treated with ESWT. Reports about treatment of nonunions in any type of allograft reconstructions with ESWT were not found in the literature, but in this patient it showed to be a valuable method. In two patients the nonunion or delayed union resulted in plate and graft fracture. Both healed after revision and augmentation with autologous spongiosa, without further delay.

The prolonged surgery duration due to the necessary harvesting of the fibula and the vascular anastomosis theoretically may increase the risk of infection, but recorded infection rates are similar to the rates when allograft alone is used [8, 14–23, 27, 29–39]. Infection in combined allograft vascularised fibula reconstructions is a severe complication, but the fibula has the ability to survive infection [30, 34, 35, 60]. In allografts alone a graft failure is seen in 8–40% (Table 1), our results reflect the advantage of a vivid bone in comparison to allograft alone especially in case of infection. In our series of twelve patients, two deep infections occurred. Unfortunately one of them died of progressive tumour disease before infection treatment could be started. In the other case, the graft could be salvaged by infection treatment with systemic antibiotics and removal of affected hardware. 

As already mentioned, the vascularised fibula expedites the achievement of a stable graft-host union and consequently leads to a reduction of the time to full unrestricted weight bearing. We allowed partial weight bearing on average 2 months after reconstruction surgery and full weight bearing at 9.4 months postoperatively, earlier than in other reported series, where full weight bearing was allowed at 14 months after operation on average (6–21) [20, 30–34, 36, 37]. To find the right balance between partial weight bearing to minimize the fracture risk and early remobilisation to increase the patient's quality of life is demanding, as literature provides no definite trend. Innocenti et al. [34] and Abed et al. [35], for example, restricted weight bearing the longest in the reviewed literature with 21.6 and 21.4 months to full weight bearing. But even in these collectives observed fracture rates of 28.5% and 36% could be seen. 

Limb length discrepancy is recorded as a complication in some reports about allograft reconstructions combined with a vascularised fibula [34–36]. In our series, however, no limb length discrepancy has been seen, but there are some patients with remaining growth, where this could still become a problem. Followup at regular intervals will be performed. 

The disadvantages, such as the prolonged surgery time with increased infection risk and the risk of anastomosis' failure by thrombosis among others, have to be kept in mind. In our series two deep infections occurred, but no anastomosis' failure. On the donor side a flexion deformity of the big toe is the most commonly observed complication. We found this complication in two patients. The effects were manageable with physiotherapy and orthotic devices. Other donor side complications recorded in the literature are pain, wound healing disorder, and ankle joint instability, and these were not observed in our patient collective [20, 31, 32, 34, 38].

Complications are quite frequent in this reconstruction type as 50% of our patients needed at least one additional surgical intervention to treat complications, and similar rates are reported in the literature. But despite this frequency, complications are manageable, and only few grafts fail. In our series we found a five-year survival rate of 100%, and in the literature midterm survival rates are also reported ranging between 80% and 100% [30, 32–39]. Capanna et al. [30] reported the series with the longest followup with 93.3% graft survival after 9 years on average, favourably better graft survival in comparison to reconstructions with allograft alone (Tables 1 and 2). Although long-term survival is rarely reported [30, 34], it can be suggested that—similar to single allograft reconstructions—survival rates will not further decline, and the construct becomes a stable and durable system.

Intercalary endoprostheses are a less popular alternative to allograft reconstructions. Published data is rare, and comparison to results with intercalary allograft reconstructions is difficult, as the patients are older in the endoprosthetic series, and also patients with metastatic disease are included [43, 46]. Endoprosthetic reconstruction offers early weight bearing and normal function on one hand, but infection and prosthetic or periprosthetic fracture as well as aseptic loosening and mechanical wear are feared complications on the other hand [9, 42, 43, 45]. Infection and fracture rates are relatively low ranging from 0 to 3.6% and from 0 to 16%, but aseptic loosening is the major problem occurring in up to 33% (Table 3) [42–46].

The major concern in the use of endoprostheses in young patients is their high potential for late failure. While allografts achieve a stable state after the first years, endoprostheses continues to fail [22, 42, 43, 46, 49, 51, 53]. Hanna et al. [42] recorded a five-year survival rate of 85% that declined to 68% at 10 years. Aldlyami et al. [45] published the series with the longest followup and reported a ten-year survival rate of 63%, and the curve is still declining, without achieving a stable plateau. The latter authors even wrote that they do not recommend intercalary endoprostheses in tibial reconstructions except in a palliative situation, but called it an attractive option in femoral defects. But as endoprostheses seem to be inferior to allografts in their durability, they should be used where immediate weight bearing and full function are of greater concern than durability, like in patients with metastatic disease.

In conclusion the presented series indicates that the combination of massive bone allograft and autologous vascularised fibula can achieve a stable and durable reconstruction, despite relatively high complication rates, as the complications are manageable. This reconstruction method is especially beneficial in young patients with primary tumours in contrast to patients with limited life expectancy, as long-term results in the reviewed literature are promising, but further investigations are necessary.

## Figures and Tables

**Table 1:** Comparison of intercalary allograft reconstructions in the lower limb.

Authors	Patients	Fracture	Infection	Nonunion	Grafts failed	Graft survival rate at 5 y/10 y [%]
Zimel et al. [15]	38	1 (2.6%)	7 (18.4%)	6 (15.8%)	15 (39.5%)	70/53
Muscolo et al. [8]	13	3 (23%)	1 (7.8%)	2 (15.4%)	4 (30.8%)	69.2% at 5.25 years
Muscolo et al. [16]	59	4	3 (5%)	9 (15.3%)	9 (15.3%)	79/—
Deijkers et al. [18]	35	12 (34.3%)	3 (8.6%)	10 (28.6%)	6 (17%)	—/79
Chen et al. [14]	13	2 (15.4%)	0	6 (46.2%)	1 (7.7%)	92.3% at 5.5 years

Percentages in brackets.

**Table 2:** Comparison of intercalary reconstructions with massive allograft and vascularised fibula in the lower limb.

Author	Patients	Complications donor site	Infection	Fracture	Nonunion	Failure	Reconstruction survival
Presented results	12	2 in 2 pat	2 (16.6%)	4 (33.3%)	4 (33.3%)	0	100% at ~3.5 years
Capanna et al. [30]	90	n.s	7 (7.5%)	12 (13.3%)	8 (8.8%)	6 (6.5%)	93.3% at ~9 years
Li et al. [32]	11	10 in 5 pat	0	0	1 (9%)	1 (9%)	90.9% at ~2.8 years
Jager et al. [33]	7	7 in 6 pat	1 (14.28%)	1 (14.28%)	2 (28.5%)	0	100% at ~3.7 years
Li et al. [31]	8	4 in 2 pat	0	0	2 (25%)	1 (12.5%)	87.5% at ~3.2 years
Innocenti et al. [34]	21	5 in 4 pat	1 (4.7%)	6 (28.5%)	2 (9.5%)	4 (19%)	80.9% at 10 years
Abed et al. [35]	25	6 in 5 pat	1 (4%)	9 (36%)	1 (4%)	5 (20%)	79% at 5 years
Moran et al. [36]	7	1 in 1 pat	0	2 (28.6%)	2 (28.5%)	0	100% at ~4.3 years
Bernd et al. [37]	16	1 in 1 pat	2 (12.5%)	1 (6.25%)	5 (31.3%)	1 (6.3%)	93.75% at ~5 years
Hennen et al. [38]	10	0	1 (10%)	0	1 (10%)	2 (20%)	80% at ~2.6 years
Ozaki et al. [39]	12	n.s	0	4 (33%)	n.s	0	100% at ~2.6 years

n.s: not specified; Percentages in brackets.

**Table 3:** Comparison of reconstructions with intercalary endoprostheses.

Author	Patients	Survival of implant	Infection (F/T/H)	Prosthetic fracture (F/T/H)	Periprostehtic fracture	Aseptic loosening (F/T/H)
Hanna et al. [42]	28	85% at 5 y and 68% at 10 y	3.6% (1/0/0)	7.1% (2/0/0)	3.60%	3.6% (1/0/0)
Ruggieri et al. [43]	24	n.s	0	4.2% (1/0/0)	0	25% (4/1/1)
Abudu et al. [44]	18	n.s	0	0	0	33% (3/1/2)
Aldlyami et al. [45]	35	63% at 10 y	2.9% (0/1/0)	5.7%	2.90%	20% (5/1/1)
Ahlmann et al. [46]	6	100% at 1 y. 83% at 2 y	0	0	0	16.7% (0/0/1)

n.s: not specified; F: femur; T: tibia; H: humerus.
